# Radar Constant-Modulus Waveform Optimization for High-Resolution Range Profiling of Stationary Targets

**DOI:** 10.3390/s17112574

**Published:** 2017-11-08

**Authors:** Wenzhen Yue, Lin Li, Yu Xin, Tao Han

**Affiliations:** Beijing Institute of Remote Sensing Information, Beijing 100192, China; thisisll@sina.com (L.L.); xinyu@mail.ustc.edu.cn (Y.X.); ihantx@163.com (T.H.)

**Keywords:** high-resolution range profiling, radar waveform optimization, constant-modulus waveform, stationary target

## Abstract

The high-resolution range (HRR) profile is an important target signature in many applications (e.g., automatic target recognition), and the radar HRR profiling performance is highly dependent on radar transmitted waveforms. In this paper, we consider the constant-modulus (CM) waveform optimization problem to improve HRR profiling performance for stationary targets. Firstly, several fundamental bounds regarding the profiling ambiguity, stability, and accuracy are derived. Further investigation reveals that the stability and accuracy of HRR profiling are unified in the white noise case. Aimed at improving the profiling stability and accuracy, we design two types of CM radar waveforms—the arbitrary-phase and QPSK waveforms—through a customized Gaussian randomization method. The performance of LFM waveforms is also discussed. Numerical experiments show that the optimized CM waveforms can dramatically enhance the profiling performance over the unoptimized ones.

## 1. Introduction

Obtaining the high-resolution range (HRR) profile (HRRP) of targets is a significant capacity of modern radars and the HRRP plays a critical role in synthetic aperture radar [1], automatic target recognition [2,3,4,5] and classification [6,7], and adaptive waveform design [8,9], etc. It has been widely acknowledged that the HRR profiling performance is associated with the waveform utilized [10], and improper waveforms can result in large profiling errors [11]. In this paper, we focus on the waveform optimization problem, aiming to improve the profiling performance for stationary targets via the optimized waveforms.

In recent decades, radar waveform design has received considerable attention. Generally speaking, the existing waveform design methods can mainly be classified into three categories. The first is information theory-based methods [12,13,14,15,16] mainly for target estimation or identification, whose aim is to acquire the most information out of targets. In this category of study, random target models are usually used, and mutual information (MI) between the received signal and the target impulse response (TIR) is maximized [12]. In [13], it was shown that the maximization of the conditional MI and the minimization of the mean square error (MSE) lead to the same solution. But the two criteria result in different waveforms when the uncertainty of the target power spectrum is considered [14]. The multiple-target case was discussed in [15]. The second category is ambiguity function (AF) -based methods [17,18,19,20], which optimize the waveforms by shaping the AF. In [17], a phase-modulated waveform was synthesized to minimize the out-of-bin clutter contribution for improved detection in heavy sea clutter. In [18], phase-modulated waveforms were designed to minimize the AF over some specific range-Doppler bins. The transmitted waveform is optimized via maximizing the AF peak for a distributed MIMO radar system in [19]. In [20], unimodular waveforms with low correlation sidelobes in one or more lag intervals are designed. The third category is the signal-to-noise ratio (SNR) -based methods [8,9,13,21,22,23,24,25,26,27], which aim to maximize the output SNR at the receiver end. Bell [13], for the first time, obtained the eigen-waveform solution under the total energy constraint. For the clutter-present case, Pillai [22] proposed the eigen-iterative algorithm to determine the optimal transmitter and receiver. Paper [8] solved the potential non-convergence problem in [22] via the alternate optimization method; reference [9] further designed the constant-modulus (CM) waveform with acceptable performance loss compared with the non-CM waveforms obtained in [8]. In the spectral domain, phase-modulated waveforms are optimized by approaching the optimal energy spectral density [23,24]. Waveforms are optimized for extend target recognition with polarimetric radar in [25,26,27]. In addition, the intrapulse radar-embedded covert communication is discussed in [28]. 

The existing work regarding waveform optimization for HRR profiling (also called target estimation in some literature) is mainly based on information theory (i.e., the first category). The assumptions of this category of work, however, are usually unrealistic (e.g., the target scattering amplitude is randomly distributed), hampering the practicability of the methods. In this study, we revisit the problem from another perspective, which is based on the HRR profiling performance analysis of the waveforms. Furthermore, to fully utilize the transmitter’s power and avoid nonlinear distortion brought by the radio-frequency amplifier, the CM constraint is imposed on transmitted waveforms—which is usually ignored in existing works. Note that the CM property is important for power-efficient radars, e.g., airborne and spaceborne radars.

In this paper, we focus on the optimization of CM phase-modulated waveforms for HRR profiling without strong assumptions on the target/environment model. Firstly, we derive the profiling unambiguity criterion, the upper and lower bounds of the profiling error, which are corresponding to the ambiguity, stability and accuracy of HRR profiling, respectively. By further analyzing these derived results, we obtain some useful conclusions and design two types of CM phase-modulated waveforms—the arbitrary-phase waveform and the quadrature phase shift keying (QPSK) waveform. The proposed waveform design method can be applied to both the white noise and the colored noise. In addition, the profiling performance of LFM waveforms in the white noise is discussed in theory and simulation.

The rest of the paper is organized as follows: a discrete baseband radar signal model is formulated in Section 2. Section 3 provides several fundamental limits of HRR profiling, including the profiling ambiguity, stability and accuracy. In Section 4, two types of CM phase-modulated waveforms are designed. Numerical results are provided, and some useful findings are obtained in Section 5. Finally, the main conclusions are drawn in Section 6.

Notation: Throughout this paper, boldface lowercase and uppercase letters represent vectors and matrices, respectively. The superscripts ${\left(\cdot \right)}^{T}$, ${\left(\cdot \right)}^{*}$, and ${\left(\cdot \right)}^{H}$ denote the transpose, conjugation and Hermitian transpose operations, respectively. $I_{n}$ is the n×n unity matrix. $0_{m\times n}$ ($0_{n}$) and $1_{m\times n}$ ($1_{n}$), respectively, are the m×n (n×1) all-zero matrix and all-one matrix. We omit the subscripts when it does not cause confusion in the matrix/vector size. $A_{mn}$ represents the element located at the *m*th row and *n*th column of A. a(n) or $a_{n}$ represents the *n*th element of a. ‖⋅‖ and ${\left\|\cdot \right\|}_{F}$ denote the 2-norm and the Frobenius norm, respectively. Re(⋅) and Im(⋅) are the real and imaginary operators, respectively. CN(0, A) represents the complex normal distribution with zero mean and covariance matrix A. Function diag(a) returns a diagonal matrix with the elements of a on the main diagonal. The notation ∗ represents the convolution operator. tr(⋅) represents the trace of a matrix. In the paper, operator ./ denotes the element-wise division, and sin() is an element-wise function.

## 2. Signal Model

**Figure 1:**
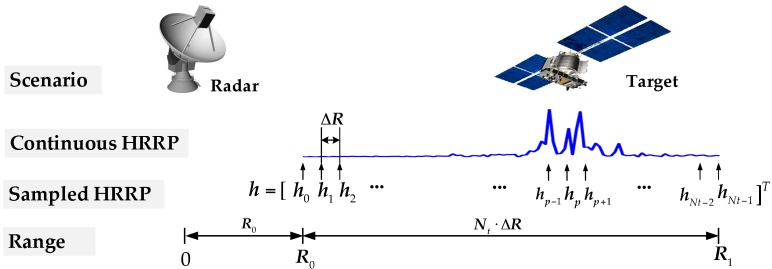
An illustration of the radar/target signal model.

In this study, we focus on the traditional single-input single-output (SISO) radar model (the MIMO radar case will be a follow-up study). Assume that the target of interest falls in the range gate $\left[R_{0},\;R_{1}\right]$. See Figure 1 for an illustration. Partition $\left[R_{0},\;R_{1}\right]$ into a series of range slices with each slice being ΔR, which is the range resolution of the radar system. ΔR is set as the radar range resolution c/2B, where *c* is the speed of light and *B* is the bandwidth. Then, the HRR profile of the target can be expressed as a vector $h≜{\left[h_{0},\;h_{1},\;\cdots h_{N_{t}-1}\right]}^{T}$, where $h_{p}$ represents the complex scattering amplitude of the scatterer located at $R_{0}+p\Delta R$. For simplicity, we assume that the path loss has been absorbed into $h_{p}$ [29]. In this paper, we only consider stationary targets, or equivalently, assume that the relative velocity between the target and the radar has been compensated.

Based on the above target model, the echoes can be expressed as:

(1) $$x_{RF}\left(t\right)=\sum_{p=0}^{N_{t}-1}h_{p}\cdot \exp\left(j2\pi f_{c}\left(t-\frac{2R_{0}}{c}-\frac{2p\Delta R}{c}\right)\right)\cdot s\left(t-\frac{2R_{0}}{c}-\frac{2p\Delta R}{c}\right)+n\left(t\right)$$

where s(t) is the baseband transmitted waveform, and n(t) is the channel noise. After the down-conversion, the received baseband signal is:

(2) $$x_{BS}\left(t\right)=\sum_{p=0}^{N_{t}-1}h_{p}\cdot \exp\left(j2\pi f_{c}\left(-\frac{2R_{0}}{c}-\frac{2p\Delta R}{c}\right)\right)\cdot s\left(t-\frac{2R_{0}}{c}-\frac{2p\Delta R}{c}\right)+n_{BS}\left(t\right)$$

where $n_{BS}\left(t\right)$ is the baseband noise. The exponential item in Equation (2) is the phase shift induced by the carrier frequency f and is only related to p, and thus can be absorbed into $h_{p}$. Therefore, Equation (2) can be simplified to:

(3) $$x_{BS}\left(t\right)=\sum_{p=0}^{N_{t}-1}h_{p}\cdot s\left(t-\frac{2R_{0}}{c}-\frac{2p\Delta R}{c}\right)+n_{BS}\left(t\right)$$

Equation (3) can be considered as the output of a linear time-invariant system whose impulse response is h and whose input signal is s(t). Then the discrete version of Equation (3) can be written as:

(4) x=s∗h+n

where $s\in {\mathbb{C}}^{N_{s}}$ is the discrete baseband transmitted waveform, $n\in {\mathbb{C}}^{N_{n}}$ is the sampled version of $n_{BS}\left(t\right)$, which obeys the complex Gaussian distribution. Equation (4) can be rewritten in matrix form as:

(5) x=Sh+n

where matrix ***S*** has the following form:

(6) $$S\;=\;\left[\begin{array}{cccc} s_{0} & 0 & \cdots & 0\\ \begin{array}{c} \vdots \\ s_{N_{s}-1}\\ 0 \end{array} & \begin{array}{c} s_{0}\\ \vdots \\ s_{N_{s}-1} \end{array} & \begin{array}{c} \ddots \\ \ddots \\ \ddots \end{array} & \begin{array}{c} \vdots \\ 0\\ s_{0} \end{array}\\ \vdots & \ddots & \ddots & \vdots \\ 0 & \cdots & 0 & s_{N_{s}-1} \end{array}\right]$$

Matrix ***S*** is termed the waveform convolution matrix (WCM), and can be written mathematically as:

(7) $$S=\left[\xi_{0}\;\cdots \;\xi_{N_{t}-1}\right],\;where\;\xi_{i}={\left[0_{i}^{T}\;s^{T}\;0_{N_{t}-i-1}^{T}\right]}^{T},\;i=0,\;\cdots ,\;N_{t}-1$$

Equation (5) gives the relation between the received baseband signal x and the waveform s, and forms the foundation of further analysis.

## 3. Performance Analysis of HRR Profiling

The performance of HRR profiling is highly dependent on the transmitted waveform. In this section, several fundamental limits regarding profiling ambiguity, stability and accuracy are discussed. The analysis results in this section can be used to assess the profiling performance of the transmitted waveform, and aid in the waveform optimization in Section 4. 

### 3.1. Unambiguous Criterion

Unambiguity is an essential requirement to make the HRR profiling result unique and applicable [10]. One can see from Equation (5) that the echo x is a linear combination of the columns of WCM ***S***. To maintain the unique estimation of h, S must be full-column rank. We then have the following theorem.

**Theorem** **1** (Unambiguous Criterion)**.** *The necessary and sufficient condition for HRR profiling to be unambiguous is:*

(8) $$\kappa \left(S\right)≜\zeta_{\max}\left(S\right)/\zeta_{\min}\left(S\right)<\infty$$

*In Equation (8),* $\zeta_{\max}\left(S\right)$ *and* $\zeta_{\min}\left(S\right)$ *are the maximum and minimum singular values of* S, *respectively. A more strict but unnecessary condition for HRR profiling to be unambiguous is:*

(9) $$\overset{⌣}{s}\left(\omega \right)\neq 0,\;for\;\forall \;\omega \in \left[-\pi ,\;\pi \right]$$

*where* $\overset{⌣}{s}\left(\omega \right)$ *is the discrete-time Fourier transform (DTFT) of* s.

The proof of Theorem 1 can be found in Appendix A. κ(S) in Equation (8) is often called the *condition number* of S. Equations (8) and (9) give the unambiguous criterion regarding the transmitted waveform s from the time domain and frequency domain, respectively. It is noteworthy that the requirements from Equations (8) or (9) are lax. In other words, it is not difficult for a waveform to satisfy Equations (8) and (9). 

### 3.2. Upper Bound of the Profiling Error

In real radar systems, noise always exists and leads to the profiling error. In this subsection, we discuss the upper bound of the profiling error, which reflects the profiling stability of the transmitted waveform. Denote:

(10) Sh=x, S(h+Δh)=x+n

where Δh represents the profiling error arising from the noise n. Theorem 2 gives the relation between the upper bound of ‖Δh‖ and the transmitted waveform s.

**Theorem** **2** (Profiling Stability)**.** *Assume that the unambiguous condition in Theorem 1 holds, i.e., WCM* S *is full-column rank. Then we have:*

(11) $$\frac{\left\|\Delta h\right\|}{\left\|h\right\|}\leq {\left[\kappa \left(S\right)\right]}^{2}\cdot \frac{\left\|n\right\|}{\left\|x\right\|}$$

*where* κ(S) *has been defined in Equation* (8). *More loosely, we have that:*

(12) $$\frac{\left\|\Delta h\right\|}{\left\|h\right\|}\leq \left\{\frac{\gamma }{\varepsilon }+\sqrt{{\left(\frac{\gamma }{\varepsilon }\right)}^{2}-1}\right\}\cdot \frac{\left\|n\right\|}{\left\|x\right\|}$$

*where* γ *and* ε *are the maximum and minimum of* $\overset{⌣}{b}\left(\omega \right)$, *respectively;* $\overset{⌣}{b}\left(\omega \right)$ *is the power spectrum density (PSD) of the transmitted waveform **s** and is defined as:*

(13) $$\overset{⌣}{b}\left(\omega \right)=\sum_{n=-N_{t}+1}^{N_{t}-1}b_{n}\exp\left(jnw\right),\;b_{n}=\sum_{k=\max\left(0,n\right)}^{\min\left(N_{s}-1,N_{s}-1+n\right)}s\left(k\right)s^{*}\left(k-n\right)$$

*Note that Equation (12) requires that* $\varepsilon =\min\left\{\overset{⌣}{b}(w)\right\}>0$*, i.e., Equation (9) holds.*

The proof of Theorem 2 can be found in Appendix B. Note that Theorem 2 is based on the unambiguity of HRR profiling. Equations (11) and (12) give two types of upper bounds of the profiling error, from the time domain and frequency domain, respectively. Equation (11) indicates that the upper bound is positively associated with κ(S); whereas Equation (12) implies that the upper bound is positively associated with the flatness of the PSD of the transmitted waveform. Also note that Equation (11) is a tighter upper bound than Equation (12) (see Appendix B for the reason). In addition, the upper bounds in Theorem 2 is irrespective of the probability distribution of noise. In other words, they apply to any form of noise distribution.

### 3.3. Lower Bound of the Profiling Error

In this subsection, we discuss the Cramer-Rao lower bound (CRB) of the profiling error, which reflects the profiling accuracy of the transmitted waveform. The discussion is based on the assumption that the noise is Gaussian distributed.

**Theorem** **3** (Profiling Accuracy)**.** *Assume that the noise* n *obeys the complex Gaussian distribution with mean value being **0** and covariance matrix* $R_{n}$*, i.e.,* $n\sim CN\left(0,\;R_{n}\right)$*. Then, for the parameter vector* θ=[ReT(h)  ImT(h)]T∈2Nt *to be estimated, the CRB matrix is:*

(14) Cθ=12⋅{Re[FHRn−1F]}−1

*where* F=[S jS]*. Therefore, the mean square error (MSE) values of an unbiased estimation* $\hat{\theta }$ *satisfies:*

(15) $$E\left\{{\left\|\hat{\theta }-\theta \right\|}_{F}^{2}\right\}\geq tr\left(C_{\theta }\right)≜C_{h}$$

Theorem 3 is the direct derivation of the Slepian-Bang’s Theorem [30]. One can refer to [30] for the proof details. Equation (15) shows that the best achievable profiling accuracy with the waveform ***s*** is $C_{h}$, the trace of matrix $C_{\theta }$. However, the expression of $C_{\theta }$ in Equation (14) is somewhat complicated. We will analyze it in the next section.

## 4. Constant-Modulus Waveform Design

Based on the profiling performance analysis about the transmitted waveform in Section 3, we focus on the CM waveform optimization problem in this section. First, we investigate the relations of the profiling ambiguity, stability, and accuracy in both white and colored noise. It is shown that their requirement on the waveforms are unified in the white noise. Then, two types of phase-modulated waveforms are designed in the sense of improving the profiling stability and accuracy. Besides, the potential of LFM waveforms for HRR profiling is also discussed. 

### 4.1. Problem Analysis

First, we consider the white noise case. Let $R_{n}=\sigma_{n}^{2}I$, where $\sigma_{n}^{2}$ is the noise power. A good waveform should make the HRR profiling unambiguous and, at the same time, exhibit good profiling stability and accuracy. Technically, (i) from the perspective of the profiling unambiguity and stability, the condition number of WCM S κ(S) should be small (see Equations (8) and (11) for the reason); (ii) from the perspective of the profiling accuracy, the CRB in Equation (14) should be as small as possible.

As for the profiling stability, it is not difficult to check that, the sufficient and necessary condition for κ(S) to achieve the minimum is that:

(16) $$B≜S^{H}S=\left(s^{H}s\right)\cdot I$$

In what follows, the discussion of the profiling accuracy is mainly focused on. For notational simplicity, we let $s^{H}s=N_{s}$. In the white noise case, the CRB matrix in Equation (14) is simplified to:

(17) Cθ=σn22⋅{Re[FHF]}−1

Because F=[S jS], we have that:

(18) Ω≜Re[FHF]=Re[SHSj⋅SHS−j⋅SHSSHS]=[Re(SHS)−Im(SHS)Im(SHS)Re(SHS)]

Because $S^{H}S$ is a positive definite Hermite matrix (S is assumed to be full column rank), matrix Ω is a real symmetric positive-definite matrix. Therefore, the eigenvalues of $S^{H}S$ and Ω are positive and real. Denote the eigenvalues of $S^{H}S$ by $\lambda_{1}\geq \lambda_{2}\geq \cdots \geq \lambda_{N_{t}}>0$. Then, $\left\{\lambda_{i},\;\lambda_{i}\right\},\;i=1,\;\cdots ,\;N_{t}$ are the eigenvalues of matrix Ω, and $\left\{1/\lambda_{i},\;1/\lambda_{i}\right\},\;i=1,\;\cdots ,\;N_{t}$ are the eigenvalues of $\Omega^{-1}$. Considering that the trace of a matrix is equal to the summation of all its eigenvalues, $C_{h}$ can be written as:

(19) $$C_{h}=tr\left(C_{\theta }\right)=\sigma_{n}^{2}\sum_{i=1}^{N_{t}}\left(1/\lambda_{i}\right)$$

For positive numbers, their arithmetic mean is not less than their harmonic mean [31]. Therefore, we have:

(20) $$\left(\sum_{i=1}^{N_{t}}\lambda_{i}\right)/N_{t}\geq N_{t}/\left(\sum_{i=1}^{N_{t}}1/\lambda_{i}\right)$$

hence:

(21) $$C_{h}=tr\left(C_{\theta }\right)=\sigma_{n}^{2}\sum_{i=1}^{N_{t}}\left(1/\lambda_{i}\right)\geq \left(\sigma_{n}^{2}N_{t}^{2}\right)/\left(\sum_{i=1}^{N_{t}}\lambda_{i}\right)$$

The equality in Equation (21) holds if and only if:

(22) $$\lambda_{1}=\cdots =\lambda_{N_{t}}$$

in which case, matrix ***B*** is a scaled identity matrix, i.e.,

(23) $$B=S^{H}S=\left(s^{H}s\right)\cdot I$$

Comparing Equations (16) and (23), we can find that in the white noise case, κ(S) and CRB achieve the minima under the same condition. It means that the requirements of the profiling unambiguity, stability, and accuracy regarding the transmitted waveform is unified. In other words, the improvement of the profiling accuracy will be accompanied by the improvement of the profiling stability. A remark is given in the following paragraph regarding the upper bound of $C_{h}$.

**Remark** **1.** *Appendix C* *gives an upper bound of* $C_{h}$ *(see (A19) in* *Appendix C* *), which is positively associated with the condition number of* S*. The upper bound of the CRB gives the worst case of the profiling accuracy. The smaller the value of* κ(S)*, the better the worst case of the profiling accuracy. This phenomenon, once again, demonstrates that the stability and accuracy of the HRR profiling is unified in the white noise case.*

Based on the preceding analysis, the quality of the transmitted waveform can be assessed by the value of κ(S), which can also be assessed by the approximation of $B=S^{H}S$ and $N_{s}\cdot I$. Next, we use the autocorrelation sequence to quantify the approximation. The structure of matrix B, which is a Toeplitz and Hermite matrix, is used. More specifically, B can be written as:

(24) $$B\;=\;\left[\begin{array}{cccc} b_{0} & b_{1} & \cdots & b_{N_{t}-1}\\ b_{-1} & \ddots & \ddots & \vdots \\ \vdots & \ddots & \ddots & b_{1}\\ b_{-N_{t}+1} & \cdots & b_{-1} & b_{0} \end{array}\right]$$

where:

(25) $$b_{n}=\sum_{k=\max\left(0,n\right)}^{\min\left(N_{s}-1,N_{s}-1+n\right)}s\left(k\right)s^{*}\left(k-n\right),\;n=-N_{t}+1,\cdots ,N_{t}-1$$

Let the first column and the first row of B form vector b:

(26) $$b={\left[b_{-Nt+1},\;\cdots ,b_{0},\cdots ,b_{Nt-1}\right]}^{T}$$

One can find that b is the autocorrelation sequence of s. 

For the best case in the white noise scenario, the ideal matrix B is the scaled identity matrix, meaning that the ideal vector b is:

(27) $$b^{t}={\left[0,\cdots ,\;0,\;N_{s},\;0\cdots ,0\right]}^{T}$$

We refer to the ideal $b^{t}$ as the autocorrelation template (AT) in our study. Therefore, the MSE between the practical autocorrelation sequence and the AT can be used to assess the transmitted waveform. We define the MSE as:

(28) $${\left\|\left(b-b^{t}\right)/N_{s}\right\|}^{2}$$

Generally speaking, the smaller the MSE, the better the transmitted waveform. 

In what follows, we derive the autocorrelation template $b^{t}$ in the colored noise case, which is more complicated than the white noise case. Since $b^{t}$ is obtained via the matrix $S^{H}S$, we need to determine the optimum $S^{H}S$ firstly. By using a similar analysis procedure, we can get that the optimum waveform satisfies:

(29) $$S^{H}R_{n}^{-1}S=a\cdot I$$

which can be regarded as a weighted version of Equation (23). In Equation (29), $a=\xi_{0}^{H}R_{n}^{-1}\xi_{0}$ is a constant value. Decompose $R_{n}$ via the Cholesky decomposition (or via eigenvalue decomposition), and obtain $R_{n}=\Gamma \Gamma^{H}$, where Γ is an invertible matrix. Then, Equation (29) turns into:

(30) $${\left(\Gamma^{-1}S\right)}^{H}\left(\Gamma^{-1}S\right)=a\cdot I$$

Therefore, $\Gamma^{-1}S=\sqrt{a}\cdot U$, where U can be an arbitrary $N_{n}\times N_{t}$ column-orthogonal matrix. Hence, $S=\sqrt{a}\Gamma U$, and:

(31) $$S^{H}S=a\cdot U^{H}\Gamma^{H}\Gamma U$$

The form of U is not unique, so the form of $S^{H}S$ is not unique as well. Most simply, let U be the matrix whose diagonal elements are 1 and the others are 0. Then Equation (31) becomes:

(32) $$S^{H}S=a\cdot {\left[\Gamma^{H}\Gamma \right]}_{\left(1:N_{t}\right):\left(1:N_{t}\right)}$$

where the subscript $\left(1:N_{t}\right):\left(1:N_{t}\right)$ denotes selecting the first $N_{t}$ rows and the first $N_{t}$ columns of $\Gamma^{H}\Gamma$. Similar with the white noise case, the autocorrelation template $b^{t}$ in the colored noise is the vector consisting of the first column and row of the new $S^{H}S$. Note that $b^{t}$ is not unique since $S^{H}S$ is not unique. 

### 4.2. CM Waveform Design

In this subsection, we use the above analysis conclusions to design CM transmitted waveform. However, obtaining the optimal CM waveform under the restrictions imposed by Equation (23) or Equation (27) is a NP-Hard problem, and the global optimum is hardly possible. Heuristic search algorithms can be used to search for a fair solution, but the computational burden is extensive and the (near) real-time processing requirement in practice can hardly be met. Next we customize the Gaussian randomization method to solve the problem, which largely decreases the computational load.

First of all, we consider the waveform s as a zero-mean stationary stochastic process Equation [9]. Namely, the correlation between elements in s is only dependent on their time difference (in discrete case, the time difference is the index difference). We then need to determine the covariance matrix of s, which is denoted as X. X is a Hermite Toeplitz matrix:

$$X_{mn}=E\left(s(m)\cdot s^{*}(n)\right)≜R_{s}(n-m)$$

For analytic convenience, in what follows we assume $N_{t}=N_{s}$. The $N_{t}\neq N_{s}$ case can be extended accordingly. In combination with Equation (25), we have:

(33) $$b_{n}=E\left(b_{n}\right)=\sum_{k=\max\left(0,n\right)}^{\min\left(N_{s}-1,N_{s}-1+n\right)}E\left[s\left(k\right)s^{*}\left(k-n\right)\right]=\left(N_{s}-\left|n\right|\right)\cdot R_{s}(n)$$

showing that the elements in $S^{H}S$ are the integer multiples of those in X. Therefore:

(34) $$X=S^{H}S./C,\;C=\left[\begin{array}{cccc} N_{s} & N_{s}-1 & \cdots & 1\\ N_{s}-1 & \ddots & \ddots & \vdots \\ \vdots & \ddots & \ddots & N_{s}-1\\ 1 & \ldots & N_{s}-1 & N_{s} \end{array}\right]$$

where ***C*** is a constant matrix.

According to Equations (23) and (34), the covariance matrix X for the white noise case is the identity matrix ***I***. Now we can use the procedures in Table 1 to design the CM arbitrary-phase waveform. The main idea is that, (i) generate a series of non-CM candidate vectors (denote the number as *K*) whose covariance matrix is X, (ii) forcibly normalize the modulus of the candidates, and (iii) among them choose the one with the minimum MSE in Equation (28). One thing worth mentioning is that, if Step 2 in Table 1 is skipped, Step 1 and 3 can be used to optimize non-CM waveforms.

Interestingly, using the method proposed in Equation [32], we can generate the QPSK waveform to realize X, which further lower the requirements upon the transmitters in comparison with the arbitrary-phase waveform. The detailed procedures for the CM QPSK waveform design are listed in Table 2. Note that Table 1 and Table 2 eliminate the heuristic search process, making it efficient to get an optimized waveform to some extent. Generally speaking, a better waveform will be obtained if the number of candidate vectors is set larger.

According to Equation (27), the ideal autocorrelation sequence of the waveform in the white noise is the impulse signal, meaning that the ideal PSD is flat. This fact suggests that LFM waveforms, which have a relatively flat PSD, should be suitable for HRR profiling. This conclusion coincides with Theorem 2 in Subsection 3.2, which says the profiling stability is related to the flatness of the PSD of the transmitted waveform. 

For the colored noise, X is not the identity matrix, but is relevant with the noise covariance matrix $R_{n}$. X can be computed via Equations (32) and (34). We can then design the arbitrary-phase or QPSK waveform via the Table 1 or Table 2. One thing worth noting is that, even though $S^{H}S$ is positive definite, X computed via Equation (34) may not be positive definite. In this case, we need to add δ⋅I to X in order to make it positive definite, where δ is the absolute value of the minimum eigenvalue of X. Additional procedures are unnecessary for Table 2, because the procedures in Table 2 can already handle the non-positive definite case.

The computational complexity of the above algorithms are related to the number of candidate vectors *K*, and the length of the waveform $N_{s}$. The details are listed in Table 3, including the CM and QPSK waveform design in both white and colored noise . The worst-case computational complexity is $O\left(K\cdot N_{s}{}^{3}\right)$. One can see that, if the pulse width of the transmitted waveform is fixed, the computational complexity is proportional to *K*. Therefore, *K* can be determined according to the computational capability of the radar system. Statistically, a larger *K* means a heavier computational burden and a better optimization result.

## 5. Simulation Results

In this section, numerical experiments are conducted to demonstrate the analyzed results and the proposed waveform design methods. The white and colored noise cases are discussed, respectively. 

### 5.1. White Noise Case

In this subsection, we compare the HRR profiling performance of several waveforms, including the monotone waveform $s_{1}$, two LFM waveforms $s_{2}$ and $s_{3}$ with different parameters, the optimized CM arbitrary-phase and QPSK waveforms $s_{4}$ and $s_{5}$, and the optimized non-CM waveform $s_{6}$ (generated via Table 1 without Step 2). Set the length of s and t as: $N_{s}=N_{t}=60$. The number of the candidate vectors in Table 1 and Table 2 is set equal to 5000. The expression of the used LFM waveform is:

(35) $$s\left(n\right)=\exp\left(j\cdot k\pi {\left(n/N_{s}\right)}^{2}\right),\;n=0,\;\cdots ,\;N_{s}-1$$

where *k* denotes the time-bandwith product (TBP) of the waveform, and should not be larger than $N_{s}$ (because the sampling frequency should be larger than the bandwidth). The TBPs for $s_{2}$ and $s_{3}$ are set as 30 and 60, respectively. One can see that the bandwith of $s_{3}$ is twice that of $s_{2}$. The HRR profiling performance is measured by $C_{h}$, which is computed by Equations. (17) and (19). The transmit power of the waveforms $s_{1}∼s_{6}$ is 1; namely, the total energy in each pulse period is $N_{s}$. The SNR is therefore $1/\sigma_{n}^{2}$.

**Figure 2:**
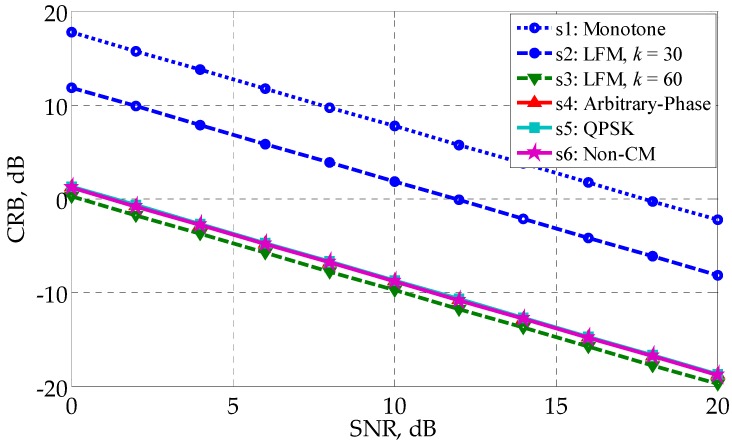
CRB versus SNR for different waveforms in the white noise.

**Figure 3:**
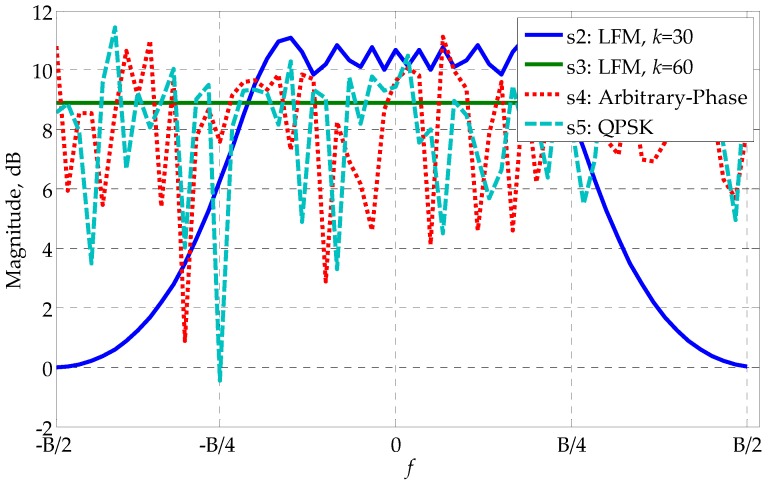
The PSDs of the waveforms $s_{2}∼s_{5}$

From Figure 2, we can see that the optimized waveforms $s_{4}∼s_{6}$ outperform the monotone waveform $s_{1}$ and the LFM waveform $s_{2}$ by more than 10 dB. It is interesting to notice that the LFM waveform $s_{3}$ with k=60 has the best profiling performance, and is slightly better than the optimized waveform $s_{4}∼s_{6}$ by roughly 0.4 dB. The reason why $s_{3}$ performs better than $s_{2}$ is that $s_{3}$ has twice the bandwidth of $s_{2}$ and larger bandwidth means better performance. (Waveforms $s_{3}∼s_{6}$ have the same bandwidth; see Figure 3 for illustration.) Figure 2 shows that LFM waveforms are naturally excellent waveforms for HRR profiling in the white noise. This finding can be considered as a newly discovered advantage of LFM waveforms. However, it should be noted that LFM waveforms could have a relatively poor performance in the colored noise since the best waveform depends on the specific noise covariance matrix. In the present example, the performance gap between the non-CM waveform $s_{6}$ and the CM waveform $s_{4}$, $s_{5}$ is negligible, even if $s_{4}$ and $s_{5}$ are generated under the CM constraint, $s_{5}$ furtherly under the QPSK constraint. It manifests the effectiveness of the customized randomization procedures presented in Table 1 and Table 2. Besides, the linear change of CRB with SNR in Figure 2 (the logarithm coordinate) can be explained by Equation (19).

Figure 3 gives the PSDs of $s_{2}∼s_{5}$. The PSD of $s_{2}$ has been shifted to align with the others. Comparing Figure 2 and Figure 3, one can see that the flatness of the waveforms corresponds to the profiling performance. $s_{3}$, with the flattest PSD, has the lowest CRB, while $s_{2}$, with the most fluctuant PSD, has the largest CRB. $s_{3}$ and $s_{4}$ perform in between $s_{2}$ and $s_{3}$, both in the flatness and the CRB. 

**Figure 4:**
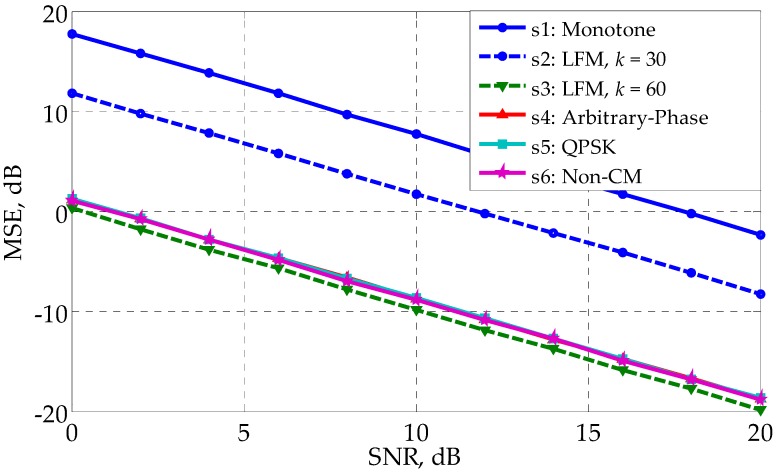
MSE (LS is used) versus SNR for different waveforms in the white noise.

Figure 4 shows that the profiling errors of $s_{1}∼s_{6}$ when the least square (LS) method is used to estimate h in Equation (5). Denote the estimated result as $\hat{h}$, and the estimation error can be expressed as:

(36) $$\mathrm{MSE}=\left(\sum_{n=1}^{N}{\left\|{\hat{h}}^{\left(n\right)}-h\right\|}_{F}^{2}\right)/N$$

where *N* is the number of independent runs and is set as 5000. Note that Equations (28) and (36) have different forms, even though they both use the concept of MSE. The results in Figure 4 coincides with those in Figure 2, with $s_{3}∼s_{6}$ better and $s_{1}$ worse. The waveform with a lower CRB has better profiling performance, demonstrating the correctness of the analysis conclusion in Section 4.1. 

**Figure 5:**
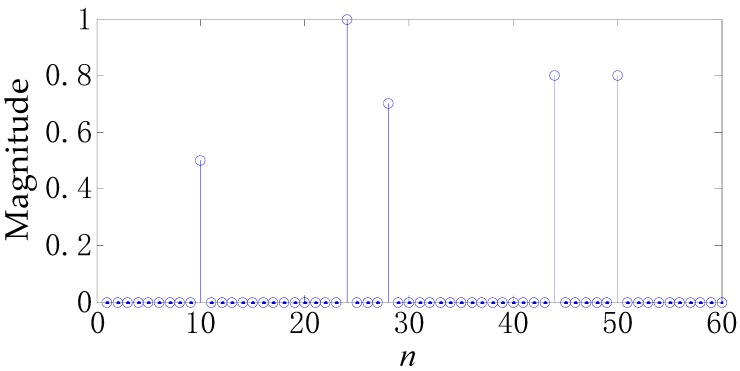
The actual target impulse response.

**Figure 6:**
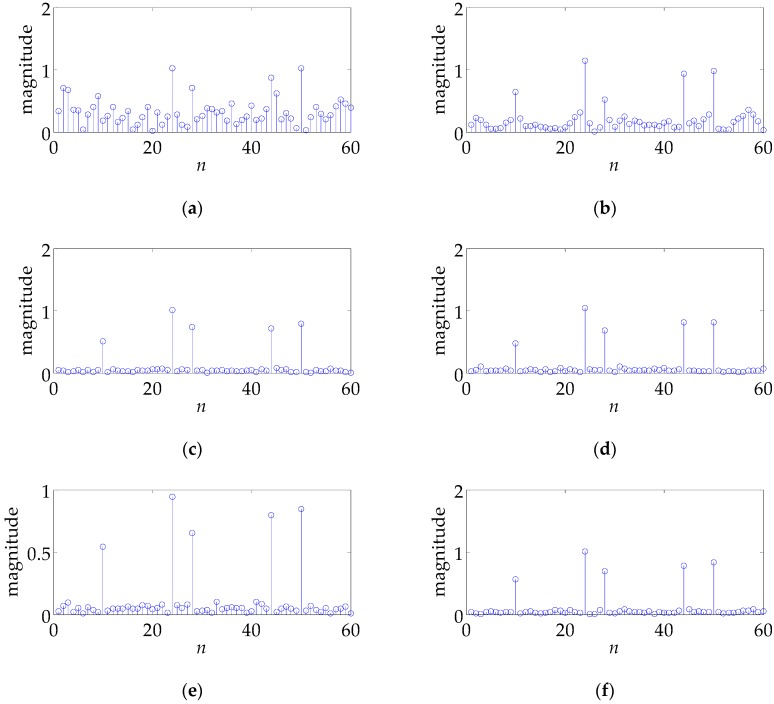
The profiling results using different waveforms in the white noise. (**a**–**f**) correspond to waveforms $s_{1}\sim s_{6}$, respectively.

In what follows, we testify the performance of $s_{1}∼s_{6}$ in a specific example. The actual TIR h is presented in Figure 5. The target consists of five major scattering points whose scattering amplitudes are set as 0.5, 1, 0.7, 0.8, 0.8, respectively. Note that the *n*-axis in Figure 5 corresponds to the range cell, which is the range resolution of the radar system. Actually, what Figure 5 shows is the radar signature of the SR-71, which has been used in [16]. Set SNR equal to 10 dB, namely, $\sigma_{n}^{2}=0.1$. Figure 6 gives the profiling results $\hat{h}$ for different waveforms. Figure 6a–f corresponds to $s_{1}∼s_{6}$, respectively. One can see that different waveforms lead to a greatly different results. $s_{3}∼s_{6}$ perform best and $s_{1}$ worst, coinciding with Figure 2 and Figure 4.

### 5.2. Colored Noise Case

In this subsection, we discuss the colored noise case. The noise covariance matrix $R_{n}$ is set as a symmetric Toeplitz matrix whose first row is $\left[1,\;q,\cdots ,q^{N_{n}-1}\right],\;q=0.5$. This setting makes $R_{n}$ positive definite. The other parameters stay the same with Subsection 5.1. Four types of CM waveforms are used, and the arbitrary-phase waveform $s_{4}$ and QPSK waveform $s_{5}$ are generated in the corresponding colored-noise way.

**Figure 7:**
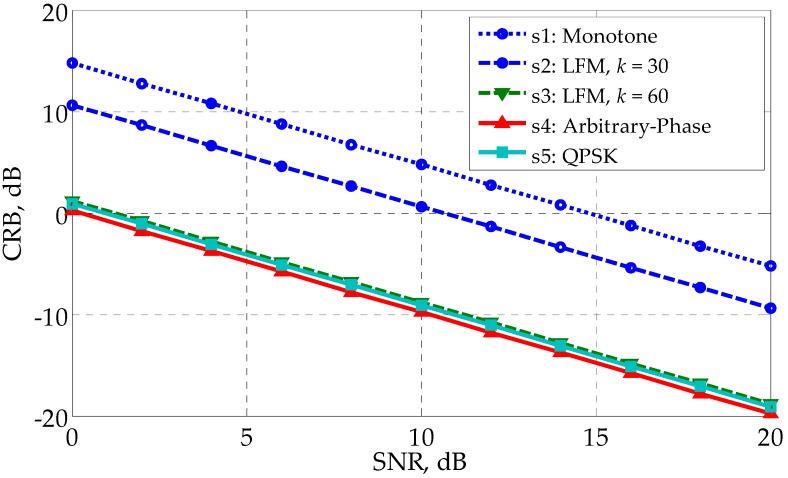
CRB versus SNR for different waveforms in the colored noise.

**Figure 8:**
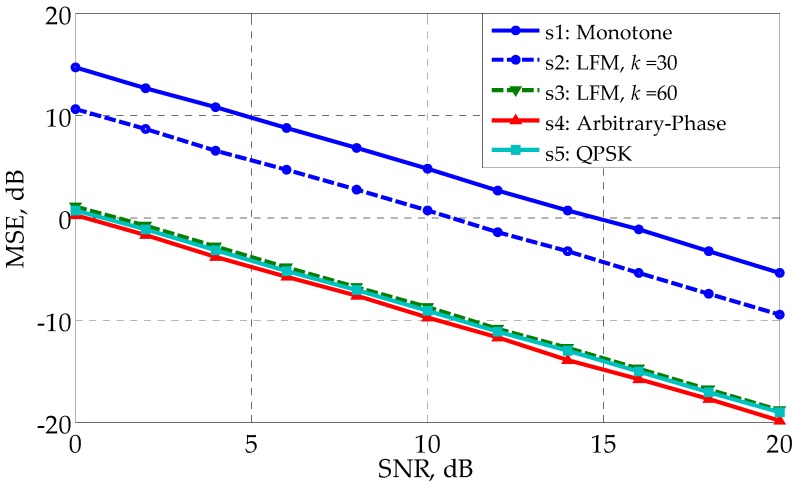
MSE (LS is used) versus SNR for different waveforms in the white noise.

Figure 7 gives the profiling performance of the waveforms in the colored noise. Comparing with Figure 2, one can see that $s_{1}\sim s_{3}$ perform worse in the colored noise than in the white noise. The CM arbitrary-phase waveform $s_{4}$ performs best, taking the place of $s_{3}$. The QPSK waveform $s_{5}$ and the LFM waveform $s_{3}$ have similar performance. Figure 8, which shows the profiling MSE of the waveforms, coincides with Figure 7.

**Figure 9:**
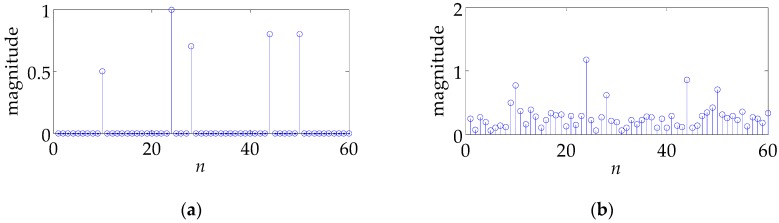

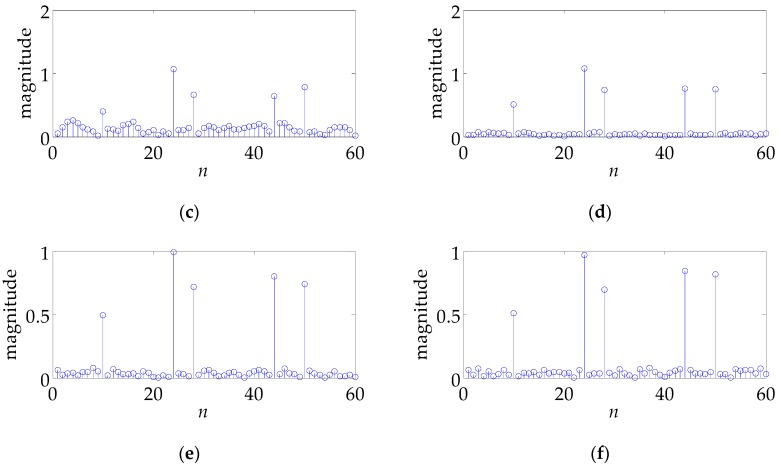
The profiling results using different waveforms in the colored noise. (**a**) is the actual TIR, and (**b**–**f**) correspond to waveforms $s_{1}\sim s_{6}$, respectively.

In the context of colored noise, the profiling results $\hat{h}$ (LS method is used) for different waveforms are shown in Figure 9. Figure 9a is the actual TIR (identical to Figure 5), while Figure 9b–f correspond to $s_{1}∼s_{5}$, respectively. The performance ranking is basically the same as that in Figure 7 and Figure 8.

## 6. Conclusions

In this paper, we consider the waveform design problem to improve the HRR profiling performance. Firstly, we derive several performance limits of HRR profiling, including the unambiguous criterion, the upper and lower bounds of the profiling error. Analysis results show that the profiling unambiguity, stability and accuracy provide the same restriction on the waveform in the white noise. In the sense of minimizing the CRB, we design two types of CM waveforms—the arbitrary-phase waveform and the QPSK waveform—through Gaussian randomization method. Numerical results demonstrate the outstanding performance of the designed CM arbitrary-phase and QPSK waveforms. LFM waveforms are also shown to have satisfactory profiling performance in the white noise, without the optimization process. This can be considered a newly discovered advantage regarding LFM waveforms. Future work will center on the extension of our conclusions herein to the moving target scenario.

## Figures and Tables

**Table 1:** Gaussian randomization method for CM arbitrary-phase waveform design.

*Step 1*: Obtain a series of non-CM Gaussian-distributed vectors (denoted as s) to realize the covariance matrix ***X***. The step can be done by means of the Cholesky factorization or eigenvalue decomposition.
*Step 2*: Get the CM vector $\bar{s}$ by normalizing the modulus of s, $\bar{s}=\exp(-j\cdot ang(s))$. $\bar{s}$ can be seen as the candidate vector for $s_{opt}$.
*Step 3:* Choose the candidate vector that minimizes Equation (28). For the white noise case, $b^{t}$ is Equation (27); for the colored noise case, $b^{t}$ is generated from $S^{H}S$ in Equation (32).

**Table 2:** Customized Gaussian randomization method for CM QPSK waveform design.

*Step 1*: Denote the real and imaginary parts of X by $X_{R}$ and $X_{I}$, respectively.
*Step 2*: Generate $\tilde{X}$ via $$\tilde{X}=\left[\begin{array}{cc} A & B^{T}\\ B & A \end{array}\right],\;A=\sin\left(\frac{\pi }{2}X_{R}\right),\;B=\sin\left(\frac{\pi }{2}X_{I}\right)$$
*Step 3*: Make a forced positive definite Cholesky decomposition $\tilde{X}+D=\Gamma \Gamma^{T}$. ***D*** is a diagonal matrix with nonnegative elements.
*Step 4*: Let β=Γθ, where θ is a Gaussian distributed vector with zero mean and unit variance. The QPSK vector can then be generated by $s=\mathrm{sgn}\left(\beta \left(1:N_{s}\right)\right)+j\cdot \mathrm{sgn}\left(\beta \left(N_{s}+1:2N_{s}\right)\right)$
*Step 5*: Generate a series of QPSK candidate vectors via the preceding steps, and choose the one that minimizes Equation (28).

**Table 3:** Computational complexity of the proposed algorithms.

Computational Complexity	CM Waveform Design	QPSK Waveform Design
White noise	$O\left(K\cdot N_{s}\right)$	$O\left(K\cdot N_{s}{}^{3}\right)$
Colored noise	$O\left(K\cdot N_{s}{}^{3}\right)$	$O\left(K\cdot N_{s}{}^{3}\right)$

## Appendix A. Proof of Theorem 1

That HRR profiling is unambiguous is equivalent to that WCM S must be full-column rank; whereas the latter is equivalent to that the condition number of S, κ(S), is finite. Therefore, Equation (8) is the sufficient and necessary condition for HRR profiling to be unambiguous. A small thing we should note is that matrix S is not square. In the square matrix case, a matrix is full-column rank is equivalent to that it is full-rank [33].

Because ℱ(⋅) is a linear and invertible transform, the linear dependency of the columns in S, ${\left\{\xi_{k}\right\}}_{k=0}^{N_{t}-1}$, is equivalent to that of ${\left\{\theta_{k}\right\}}_{k=0}^{N_{t}-1}$, where $\theta_{k}=ℱ\left(\xi_{k}\right)$. Notice that $\xi_{k},\;k=1,\;\cdots ,\;N_{t}-1$ can be obtained via the circular shift of $\xi_{0}$. Therefore, we have

(A1) $$\theta_{k}=\left[\exp{\left(-j\cdot \frac{2\pi }{N_{n}}\cdot n\cdot k\right)}_{n=0}^{N_{n}-1}\right]⊙\theta_{0}=\Pi \theta_{0},\;where\;\Pi =diag\left(\left[\exp{\left(-j\cdot \frac{2\pi }{N_{n}}\cdot n\cdot k\right)}_{n=0}^{N_{n}-1}\right]\right)$$

where ⊙ is the Hadamard product operator. Denote

(A2) $$A={\left[A_{mn}\right]}_{N_{n}\times N_{t}},\;A_{mn}=\exp\left(-j\cdot \frac{2\pi }{N_{n}}\cdot m\cdot n\right)$$

and let $\chi \in {\mathbb{C}}^{N_{t}}$ be any $N_{t}\times 1$ vector, then we have

(A3) $$\left[\theta_{0},\;\cdots ,\;\theta_{N_{t}-1}\right]\chi =\theta_{0}⊙A\chi =diag\left(\theta_{0}\right)\cdot A\chi$$

Obviously, matrix A is full column rank. In fact, $A\chi =ℱ\left(\overset{⌣}{\chi }\right)$, where $\overset{⌣}{\chi }={\left[\chi^{T}\;0_{N_{s}-1}{}^{T}\right]}^{T}$. Therefore, Aχ is an all-zero vector if and only if χ is an all-zero vector. Then, we have the following equivalent expressions.

(a) S is full-column rank.
(b) χ has to be a zero vector to make $\left[\theta_{0},\;\cdots ,\;\theta_{N_{t}-1}\right]\chi =0$, i.e., $diag\left(\theta_{0}\right)\cdot A\chi =0$.
(c) Aχ has to be an all-zero vector to make $diag\left(\theta_{0}\right)\cdot A\chi =0$.
(d) Matrix $diag\left(\theta_{0}\right)$ is full-rank.
(e) $\theta_{0}\left(n\right)\neq 0,\;for\;n=0,\;\cdots ,\;N_{n}-1$. In other words, there is no zero element in the vector $\theta_{0}$.

More strictly but unnecessarily, if the DTFT of s, $\overset{⌣}{s}\left(\omega \right)$, satisfies

(A4) $$\overset{⌣}{s}\left(\omega \right)\neq 0,\;for\;\forall \;\omega \in \left[-\pi ,\;\pi \right]$$

i.e., there is no chance for $\overset{⌣}{s}\left(\omega \right)$ to be zero, then there will be no zero element in $\theta_{0}$, either (because $\theta_{0}$ consists of the samples of $\overset{⌣}{s}\left(\omega \right)$). Therefore, Theorem 1 is proven.

## Appendix B. Proof of Theorem 2

According to Equation (10), we have that Sh=x and $\Delta h=S^{†}n$, where matrix $S^{†}$ is the Moore-Penrose inverse of WCM S. Combining with the properties of matrix norm, we can deduce that

(A5) $$\left\|h\right\|\geq \frac{\left\|x\right\|}{\left\|S\right\|},\;\left\|\Delta h\right\|\leq \left\|S^{†}\right\|\cdot \left\|n\right\|$$

Because both of the two inequalities in (A5) are positive, the division of them yields that

(A6) $$\frac{\left\|\Delta h\right\|}{\left\|h\right\|}\leq \left\|S^{†}\right\|\cdot \left\|S\right\|\cdot \frac{\left\|n\right\|}{\left\|x\right\|}$$

A similar equation has been obtained in [10] (Lemma 4.1 in [10]), whose difference from (A.6) is that the matrix therein is square while S herein is not. Because S is full column rank, $S^{†}={\left(S^{H}S\right)}^{-1}S^{H}$. Therefore,

(A7) $$\left\|S^{†}\right\|=\left\|{\left(S^{H}S\right)}^{-1}S^{H}\right\|\leq \left\|{\left(S^{H}S\right)}^{-1}\right\|\cdot \left\|S^{H}\right\|=\frac{\zeta_{\max}\left(S\right)}{{\left[\zeta_{\min}\left(S\right)\right]}^{2}}$$

where the last equality utilizes the two following equations:

(A8) $$\left\|{\left(S^{H}S\right)}^{-1}\right\|=\frac{1}{{\left[\zeta_{\min}\left(S\right)\right]}^{2}}\;and\;\left\|S^{H}\right\|=\left\|S\right\|=\zeta_{\max}\left(S\right)$$

Based on Equations (A7) and (A8), we can further obtain that

(A9) $$\left\|S^{†}\right\|\cdot \left\|S\right\|\leq {\left[\frac{\zeta_{\max}\left(S\right)}{\zeta_{\min}\left(S\right)}\right]}^{2}={\left[\kappa \left(S\right)\right]}^{2}$$

The combination of (A.6) and (A.9) leads to Equation (11). Next, we prove Equation (12). For descriptive simplicity, let $B=S^{H}S$. Then it is easily checked that matrix ***B*** is a Toeplitz matrix (see Equation (24)), and the vector consisting of ***B***’s first column and row, $b={\left[b_{-Nt+1},\;\cdots ,b_{0},\cdots ,b_{Nt-1}\right]}^{T}$, is the autocorrelation sequence of ***s***. According to the Brown and Halmos’ theorem (Theorem 2.2 in [34]), we have

(A10) $$\kappa \left(S^{H}S\right)\leq \left(\frac{\gamma }{\varepsilon }+\sqrt{{\left(\frac{\gamma }{\varepsilon }\right)}^{2}-1}\right)$$

where γ and ε are the maximum and minimum of $\overset{⌣}{b}\left(\omega \right)$, respectively. Note that Equation (A10) needs that $\varepsilon =\min\;\left\{\overset{⌣}{b}\left(\omega \right)\right\}>0$. Considering that $\overset{⌣}{b}\left(\omega \right)$ is the PSD of s, hence $\overset{⌣}{b}\left(\omega \right)\geq 0$. Furthermore, if $\overset{⌣}{s}\left(\omega \right)\neq 0$, $\overset{⌣}{b}\left(\omega \right)>0$ and ε>0. Because $\kappa \left(S^{H}S\right)={\left[\kappa \left(S\right)\right]}^{2}$, an upper bound of κ(S) can be written as

(A11) $$\kappa \left(S\right)\leq {\left(\frac{\gamma }{\varepsilon }+\sqrt{{\left(\frac{\gamma }{\varepsilon }\right)}^{2}-1}\right)}^{1/2}$$

Substituting Equation (A11) back to Equation (11) yields Equation (12). Therefore, Theorem 2 is proven.

## Appendix C. Derivation of an Upper Bound of the CRB

For descriptive simplicity, we use κ to denote the condition number of WCM S. Still denote $\lambda_{1}\geq \lambda_{2}\geq \cdots \geq \lambda_{N_{t}}>0$ as the eigenvalues of $S^{H}S$. Then these eigenvalues would satisfy

(A12) $$\sum_{i=1}^{N_{t}}\lambda_{i}=N_{s}N_{t},\;\frac{\lambda_{i}}{\lambda_{j}}\leq \kappa^{2},\;\lambda_{i}>0$$

where the second constraint condition comes from the definition of the condition number. The upper bound of $C_{h}=tr\left(C_{\theta }\right)$ can be obtained by solving the following optimization problem

(A13) $$\max\left\{\sum_{i=1}^{N_{t}}\frac{1}{\lambda_{i}}\right\},\;s.t.\;\sum_{i=1}^{N_{t}}\lambda_{i}=N_{s}N_{t},\;\frac{\lambda_{i}}{\lambda_{j}}\leq \kappa^{2},\;\lambda_{i}>0$$

Denote $S≜\sum_{i=1}^{N_{t}}1/\lambda_{i}$, yielding

(A14) $$S\cdot \sum_{i=1}^{N_{t}}\lambda_{i}=\left(\sum_{i=1}^{N_{t}}1/\lambda_{i}\right)\cdot \left(\sum_{i=1}^{N_{t}}\lambda_{i}\right)=N_{t}+\sum_{i=1}^{N_{t}}\sum_{j>i}^{N_{t}}\left(\frac{\lambda_{i}}{\lambda_{j}}+\frac{\lambda_{j}}{\lambda_{i}}\right)$$

According to Equation (A12) and the properties of function f(x)=x+1/x, x>0, we have that

(A15) $$1\leq \lambda_{i}/\lambda_{j}\leq \kappa^{2},\;1\leq i<j\leq N_{t}$$

and hence

(A16) $$2\leq \frac{\lambda_{i}}{\lambda_{j}}+\frac{\lambda_{j}}{\lambda_{i}}\leq \kappa^{2}+\frac{1}{\kappa^{2}}$$

Therefore,

(A17) $$S\cdot \left(\sum_{i=1}^{N_{t}}\lambda_{i}\right)\leq N_{t}+\sum_{i=1}^{N_{t}}\sum_{j>i}^{N_{t}}\left(\kappa^{2}+\frac{1}{\kappa^{2}}\right)=N_{t}+\frac{N_{t}\left(N_{t}-1\right)\left(1+\kappa^{4}\right)}{2\kappa^{2}}$$

By dividing both sides of Equation (A17) by $\left(\sum_{i=1}^{N_{t}}\lambda_{i}\right)$ (i.e., $N_{s}N_{t}$), we obtain

(A18) $$S\;=\;\sum_{i=1}^{N_{t}}\frac{1}{\lambda_{i}}\leq \frac{1}{N_{s}}\left(1+\frac{\left(N_{t}-1\right)\left(1+\kappa^{4}\right)}{2\kappa^{2}}\right)$$

Therefore, the upper bound of $C_{h}$ can be written as

(A19) $$C_{h}=\sigma_{n}^{2}\cdot \left(\sum_{i=1}^{N_{t}}\frac{1}{\lambda_{i}}\right)\leq \frac{\sigma_{n}^{2}}{N_{s}}\cdot \left(1+\frac{\left(N_{t}-1\right)\left(1+\kappa^{4}\right)}{2\kappa^{2}}\right)$$
